# β‐Nicotinamide Mononucleotide Inhibits Cellular Senescence in Mouse C2C12 Skeletal Muscle Cells

**DOI:** 10.1155/jare/4970524

**Published:** 2026-09-22

**Authors:** Jing Mi, Chenxin Wang, Hongfei Gu, Mengxuan Wang, Weihua Zhang, Qiangqiang Zhou, Yuejiao Zhang, Jie Pan, Liming Yu

**Affiliations:** ^1^ Department of Orthodontics, Shanghai Stomatological Hospital & School of Stomatology, Fudan University, Shanghai, China, fudan.edu.cn; ^2^ Shanghai Key Laboratory of Craniomaxillofacial Development and Diseases, Shanghai Stomatological Hospital, Shanghai, China; ^3^ Department of Stomatology, Linyi Third People’s Hospital, Linyi, China

**Keywords:** β-nicotinamide mononucleotide, cellular senescence, D-galactose, nicotinamide adenine dinucleotide

## Abstract

β‐Nicotinamide mononucleotide (NMN), as the precursor of nicotinamide adenine dinucleotide (NAD^+^), has remarkable therapeutic potential in aging‐associated diseases. Skeletal muscle exhibits not only reduced mass but also impaired strength and function during aging. However, the protective effects of NMN against skeletal muscle cell senescence remain to be fully elucidated. In this study, we used D‐galactose (D‐gal) to establish a senescence model in C2C12 cells. Our results demonstrate that aging C2C12 cells displayed a significant impairment in differentiation capacity, an effect that was ameliorated by NMN treatment, as indicated by an increase in myotube formation and elevated myosin heavy chain (MHC) expression. NMN significantly decreased reactive oxygen species (ROS) levels in both aged C2C12 myoblasts and myotubes. NMN treatment downregulated the mRNA expression of aging‐associated markers, including *tumor protein p53* (*Trp53*), *poly (ADP-ribose) polymerases* (*PARPs*), and *interleukin-1α* (*IL-1α*) in aged C2C12 myotubes. Immunofluorescence staining revealed that the expressions of p53 and interleukin‐1β (IL‐1β) were significantly upregulated in aged C2C12 myotubes, while NMN treatment significantly attenuated this elevation. Collectively, the present study demonstrates that NMN treatment attenuates D‐gal–induced ROS accumulation, enhances myotube formation, and downregulates several senescence‐associated molecules. Therefore, our findings suggest that NMN represents a promising therapeutic agent for mitigating skeletal muscle cell aging.

## 1. Introduction

Population aging is an inevitable result of population transformation and an important issue facing human society in the 21st century. By 2035, the elderly population in China is expected to reach 410 million [1], which will bring a heavy health and economic burden. Aging‐related diseases, such as sarcopenia, obstructive sleep apnea (OSA), and chronic obstructive pulmonary disease (COPD), seriously affect physical structure and function [2].

Aging is defined as the progressive functional deterioration of multiple organs and systems with advancing age. Aging of the skeletal muscular system gives rise to diminished muscle strength, which not only manifests as a reduction in muscle volume but also indicates impairments in motor function, metabolic health, and overall bodily vitality. Its hazards include an increased risk of falls and fractures, cognitive impairment, diminished quality of life, and even an elevated risk of mortality [3, 4]. Accumulating evidence indicates that the aging of skeletal muscle is driven by a complex interplay of factors, including intrinsic cellular dysfunction, perturbations in the external microenvironment, and insufficient mechanical loading [5, 6]. At the cellular level, dysregulation of redox homeostasis serves as both a vital trigger and a hallmark pathological characteristic of aging [7]. Nicotinamide adenine dinucleotide (NAD^+^) is a critical coenzyme involved in redox reactions. Cellular NAD^+^ levels gradually decline with aging, and restoring NAD^+^ levels can alleviate or even reverse many aging‐associated diseases [8]. Nevertheless, it remains elusive whether NAD^+^ supplementation in skeletal muscle cells can suppress cellular senescence.

β‐Nicotinamide mononucleotide (NMN) is a vital endogenous antiaging molecule in humans. Serving as a direct precursor of NAD^+^, NMN participates in cellular redox homeostasis and exerts a critical regulatory role in intracellular redox reactions [9]. Intracellular NAD^+^ content declines gradually with age, and NAD^+^ cannot be absorbed by the body owing to its large molecule weight [10]. NMN can effectively accelerate the biosynthesis rate of NAD^+^, suppress the excessive generation of reactive oxygen species (ROS), and alleviate mitochondrial structural damage and functional impairment. Thus, NMN exerts definite adjuvant therapeutic effects on a series of aging‐related chronic diseases, including cardiovascular and cerebrovascular diseases, diabetes mellitus, and neurodegenerative disorders [11, 12]. Additionally, NMN exerts antiaging effects on skeletal muscle by regulating capillary density and improving mitochondrial–nuclear protein imbalance in skeletal muscle, although the specific mechanisms are not fully elucidated [13].

Muscle tissue consists of muscle fibers, as well as the adjacent connective tissue, blood vessels, and nerves. In brief, skeletal myogenesis in vivo originates from muscle stem cells, which are also known as satellite cells. The satellite cells proliferate and differentiate into myoblasts upon activation. Subsequently, myoblasts undergo induced differentiation and fuse to form myotubes, which eventually mature into muscle fibers [14]. In vitro, when cultured in differentiation‐inducing medium, myoblasts exit the cell cycle—a process regulated by myogenic regulatory factors such as MyoD, and gradually elongate [15]. Upon reaching an appropriate confluence, myoblasts fuse and form multinucleated myotubes and begin to express high levels of myosin heavy chain (MHC), a key marker of myogenic differentiation [16]. As differentiation proceeds, MHC assembles with actin to form myofibrils with characteristic striations, eventually generating mature myotubes into skeletal muscle fibers in vivo [17].

In this study, we employed the protocol established by Tian et al. [18] to establish a D‐galactose (D‐gal)–induced skeletal muscle cell senescence model, subjected the established models to NMN treatment, and evaluated changes in senescence‐associated phenotypes. This study is designed to provide reliable experimental evidence for the therapeutic potential of NMN in mitigating aging‐related skeletal muscle cell senescence.

## 2. Materials and Methods

### 2.1. Materials and Reagents

Fetal bovine serum (FBS), horse serum (HS), 0.25% trypsin solution, and high‐glucose Dulbecco’s Modified Eagle Medium (DMEM) were purchased from Gibco, Thermo Fisher Scientific (Waltham, MA, USA). D‐gal (Cat. No. G0750) was obtained from Sigma‐Aldrich (St. Louis, MO, USA). NMN (Cat. No. HY‐F0004) was acquired from MedChemExpress (Monmouth Junction, NJ, USA). Total RNA was reverse‐transcribed into complementary deoxyribonucleic acid (cDNA) using the FastQuant RT Kit (TIANGEN Biotech, Beijing, China, Cat. No. KR106). Quantitative real‐time PCR (qPCR) was performed with SuperReal PreMix Plus (TIANGEN Biotech, Beijing, China, Cat. No. FP205) on a real‐time PCR system. SA‐β‐gal staining kit (Cat. No. C0602), ROS Assay Kit (Cat. No. S0033), and Cell Counting Kit‐8 (CCK‐8) Assay Kit (Cat. No. C0037) were obtained from Beyotime Biotechnology (Shanghai, China). Information on the antibodies used in this study is shown in Table 1.

**TABLE 1:** Antibody information.

Antibodies	Catalog number (cat no.)	Company	Source	Dilution ratio
MHC	MF‐20	Developmental Studies Hybridoma Bank	Mouse	1:500
p53	SC‐71820	Santa Cruz	Mouse	1:500
IL‐1β	12242	Cell Signaling Technology	Mouse	1:500
β‐Actin	abs132001	Absin	Rabbit	1:5000

### 2.2. Cell Culture

The mouse C2C12 myogenic cell line was obtained from the Cell Resource Center, Shanghai Institutes for Biological Sciences, Chinese Academy of Sciences. The cells were cultured in 6‐well plates at 37°C in an atmosphere of 5% carbon dioxide (CO_2_) using growth medium (DMEM containing 10% FBS and 1% penicillin). For myogenic differentiation, C2C12 myoblasts were induced by switching to differentiation medium (DMEM containing 2% HS and 1% penicillin) when cells reached 90% confluence. The medium was replaced every 2 days, and after 7 days of myogenic differentiation, the formation of abundant myotubes was observed under a microscope. Following the method of Tian et al. [18, 19] with minor modifications, D‐gal was dissolved in serum‐free DMEM and diluted to an appropriate concentration for later use.

After myotube formation, the following groups were established and subjected to the following treatment protocols:

a. Negative control (NC) group: This group served as the untreated control, and incubation was halted after 72 h.
b. D‐gal group: D‐gal solution was added to the culture medium, and cells were incubated for 72 h before harvesting.
c. D‐gal + NMN group: Cells were pretreated with NMN solution for 24 h, followed by medium replacement. Fresh D‐gal and NMN solutions were then coadministered, and the experiment was concluded 48 h later.

### 2.3. CCK‐8 Assay for Cell Viability Detection

C2C12 myoblasts with robust growth were collected for cell counting. Six replicate wells were prepared for each group, with 4000 cells seeded per well in 96‐well plates. After the cells had attached to the plate, they were treated with different concentrations of D‐gal according to the experimental groups. Subsequently, the cells were cultured for 12, 24, and 48 h, respectively. The CCK‐8 reagent was then added, and the plate was incubated in a 37°C incubator containing 5% carbon dioxide (CO_2_) for 60 min. The CCK‐8 cell viability assay was conducted using a multifunctional microplate reader, and the optical density (OD) values of each group were measured at a wavelength of 450 nm (BioTek). OD readings were exported, and cell viability rates were calculated using Microsoft Excel software. Statistical analyses were performed using GraphPad Prism 9.5.

### 2.4. SA‐β‐Gal Staining

After 7 days of myogenic differentiation of C2C12 cells, D‐gal was added at concentrations of 0, 2.5, 5, 10, 20, and 40 mg/mL. Following 48 h of incubation, the medium was aspirated, cells were washed with phosphate‐buffered saline (PBS), and fixed with β‐galactosidase staining fixative at room temperature for 15 min. After aspirating the fixative, the cells were washed with PBS three times, with each wash lasting 3 min. The staining solution was then added directly to the cells, which were subsequently incubated overnight at 37°C in a sealed container. Cells were visualized and imaged under a Leica microscope, and the proportion of SA‐β‐gal–positive cells was quantified.

### 2.5. Measurement of ROS Content

Briefly, the ROS detection working solution was diluted with serum‐free medium. After aspirating the cell culture medium, the cells were washed three times with PBS for 3 min each. Then, 1.5 mL of the ROS detection working solution was added to each well, and the cells were incubated in a 37°C incubator with 5% CO_2_ for 30 min. Post incubation, the cells were washed three times with DMEM. Fluorescence images were captured under a microscope, and the mean green fluorescence intensity was quantified using ImageJ software.

### 2.6. Real‐Time qPCR

Total RNA was isolated from cells using Trizol reagent, and 2 μg of RNA was reverse‐transcribed into cDNA to obtain a final 20 μL reaction system. Gene‐specific primers were designed for target genes, and the detailed sequences are listed in Table 2. A total 10 μL qPCR amplification mixture was assembled with 1 μL cDNA and gene‐specific primers. All samples were run in technical triplicate, with 18S rRNA as the internal reference gene. The experimental results were statistically analyzed using the 2^−ΔΔ*C*^_*T*_ method.

**TABLE 2:** Primer sequences for PCR amplification.

Gene	Forward (5′‐3′)	Reverse (5′‐3′)
IL‐15	CATCCATCTCGTGCTACTTGTG	GCCTCTGTTTTAGGGAGACCT
IL‐1α	TCTATGATGCAAGCTATGGCTCA	CGGCTCTCCTTGAAGGTGA
p53	CCCCTGTCATCTTTTGTCCCT	AGCTGGCAGAATAGCTTATTGAG
PARP1	ACTCATGCTACCACGCACAA	CTTTGACACTGTGCTTGCCC
PARP2	AGCTGGGAAAGACCAATCTCC	GCTGGCAGCATAGTCCATCT
18S	GTAACCCGTTGAACCCCATT	CCATCCAATCGGTAGTAGCG

### 2.7. Immunofluorescence Assay

After aspirating the culture medium from the target cells in the culture dishes, the cells were washed three times with precooled PBS. Paraformaldehyde fixative was then added, and the cells were incubated at room temperature for 15 min. After three additional PBS rinses, cell permeabilization was performed using Triton X‐100. The cells were blocked with 5% bovine serum albumin (BSA) at room temperature for 30 min. After further washing, the primary antibody was added for overnight incubation at 4°C. The next day, after washing, the goat anti‐mouse IgG secondary antibody (Cat. No. A32723, Thermo Fisher Scientific, USA) and 4′‐6‐diamidino‐2‐phenylindole (DAPI) were added for incubation. Finally, the cells were observed and photographed using an inverted fluorescence microscope (Leica).

### 2.8. Statistical Analysis

All experiments were conducted with at least three independent replicates. The images were processed and analyzed using ImageJ, and the data (mean ± standard deviation) were analyzed with GraphPad Prism. We employed one‐way analysis of variance (ANOVA) followed by Tukey’s post hoc test for comparisons among multiple groups. The asterisks ^∗^ *p* < 0.05; ^∗∗^ *p* < 0.01; and ^∗∗∗^ *p* < 0.001 indicate statistically significant differences, while ns (*p* ≥ 0.05) indicates no statistically significant difference.

## 3. Results

### 3.1. Cellular Senescence Model Was Established Using D‐Gal Treatment

**FIGURE 1:**
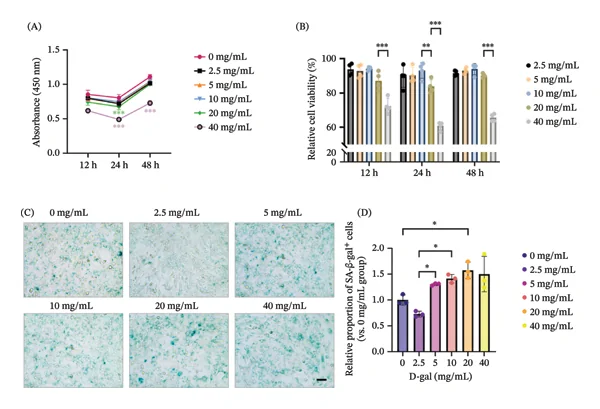
D‐gal treatment induces cellular senescence in C2C12 myoblasts. (A) CCK‐8 cell viability assay of C2C12 myoblasts. (B) Cellular viability of C2C12 myoblasts incubated with gradient concentrations of D‐gal for 12, 24, and 48 h. (C) SA‐β‐gal staining of D‐gal–treated C2C12 myotubes. Scale bars = 100 μm. (D) Quantitative statistics of SA‐β‐gal–positive cells. All experiments were performed in triplicate, with three random visual fields captured per sample. All data are presented as mean ± SD; ns, *p* ≥ 0.05; ^∗^ *p* < 0.05; ^∗∗^ *p* < 0.01; ^∗∗∗^ *p* < 0.001.

D‐gal treatment is a widely used strategy for establishing in vitro cellular senescence models [19, 20]. To investigate the appropriate concentration of D‐gal, C2C12 myoblasts were treated with different concentrations of D‐gal (0, 2.5, 5, 10, 20, and 40 mg/mL). Following D‐gal treatment for 12, 24, and 48 h, cell viability was assessed via the CCK‐8 assay (Figure 1A,B). The results demonstrated that treatment with 40 mg/mL D‐gal significantly decreased cell viability at all tested time points. Furthermore, 20 mg/mL D‐gal also induced a notable reduction in cell viability at 12 and 48 h post‐treatment.

SA‐β‐gal staining enables the visualization of senescent cells. According to the procedures described in Section 2.2., cells were cultured and treated with D‐gal and NMN, followed by senescence‐associated β‐galactosidase staining. The results showed that the proportion of SA‐β‐gal–positive cells in the 2.5 mg/mL D‐gal treatment group was slightly lower than that of the control group. With the increase in D‐gal concentration, the proportion of SA‐β‐gal–positive cells gradually increased, and the difference between the 20 mg/mL group and the control group was the most significant (Figure 1C,D). Based on the results of cell viability tests and SA‐β‐gal staining, D‐gal at 20 mg/mL could induce a sufficient number of SA‐β‐gal^+^ cells while exerting minimal impact on cell viability. Therefore, a concentration of 20 mg/mL D‐gal was selected for establishing the cellular senescence model in this study.

### 3.2. NMN Protected the Myogenic Differentiation Capacity in D‐Gal–Treated C2C12 Myotube Cells

To investigate the effect of NMN on myogenic differentiation in D‐gal–induced senescent C2C12 cells, C2C12 myotubes were pretreated with various concentrations of NMN (0, 100, 300, 500, and 800 μM) for 24 h. Subsequently, the culture medium was replaced with fresh medium containing both D‐gal and the corresponding concentration of NMN, and cells were cultured for an additional 48 h before harvest.

**FIGURE 2:**
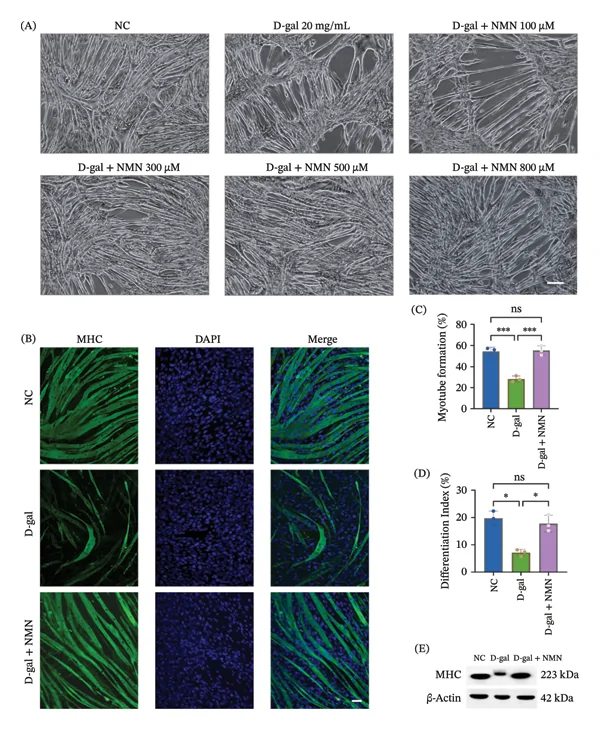
NMN alleviates the impairment of differentiation capacity in D‐gal–treated C2C12 myotubes. (A) Brightfield observation of alterations in myotube number and size after NMN treatment in C2C12 myotubes. (B) MHC immunofluorescence staining of C2C12 myotubes after D‐gal and NMN treatment. (C) Quantitative analysis of myotube formation rate based on MHC immunofluorescence staining. (D) Quantification of myogenic differentiation based on MHC immunofluorescence staining. (E) Western blot analysis of MHC protein expression. Scale bars = 100 μm. All experiments were performed in triplicate, with three random fields captured per sample. All data are presented as mean ± SD; ns, *p* ≥ 0.05; ^∗^ *p* < 0.05; ^∗∗^ *p* < 0.01; ^∗∗∗^ *p* < 0.001.

The results demonstrated that D‐gal treatment significantly inhibited myogenic differentiation of C2C12 myotubes, leading to reduced myotube formation and sparse myotube structure (Figure 2A). Treatment with 100 μM NMN exerted no obvious protective effect against D‐gal–induced myotube impairment, whereas NMN at concentrations above 300 μM observably increased both the number and diameter of fused myotubes (Figure 2A). Accordingly, 300 μM NMN was adopted for pretreatment in all subsequent experiments.

Immunofluorescence staining revealed that D‐gal markedly downregulated the expression of MHC, a key marker of myogenic differentiation, and significantly decreased both the myogenic differentiation index and myotube formation. In contrast, pretreatment with NMN significantly restored MHC expression in D‐gal–induced senescent myotubes (Figure 2B–D). Western blot analysis confirmed a consistent alteration pattern at the protein level, further verifying that NMN significantly reversed the D‐gal–induced downregulation of MHC expression (Figure 2E).

### 3.3. NMN Downregulated ROS Level Induced by D‐Gal

Elevated intracellular ROS levels are one of the characteristics of aging [21]. To investigate the effect of NMN on ROS production in D‐gal‐induced senescent C2C12 cells, cellular ROS signals were detected using a ROS fluorescent detection kit.

**FIGURE 3:**
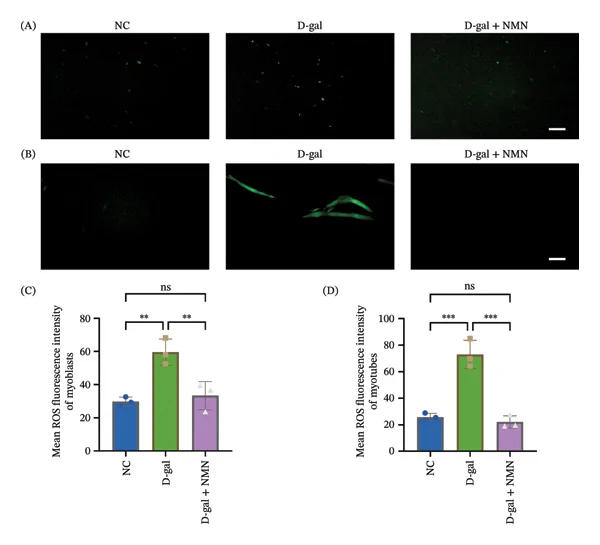
Effects of NMN and D‐gal treatment on intracellular ROS in C2C12 cells. (A) Representative images of intracellular ROS staining in C2C12 myoblasts. (B) Representative images of intracellular ROS staining in C2C12 myotubes. (C) Quantification of mean ROS fluorescence intensity in C2C12 myoblasts. (D) Quantification of mean ROS fluorescence intensity in C2C12 myotubes. Scale bars = 100 μm. All values were normalized to the NC group. All experiments were performed in triplicate, with three random visual fields captured per sample. All data are presented as mean ± SD; ns, *p* ≥ 0.05; ^∗^ *p* < 0.05; ^∗∗^ *p* < 0.01; ^∗∗∗^ *p* < 0.001.

First, intracellular ROS signals in undifferentiated C2C12 myoblasts were detected. The results revealed that D‐gal markedly elevated intracellular ROS levels in myoblasts, while NMN significantly reversed the D‐gal–induced ROS accumulation (Figure 3A,C). Subsequently, ROS signals in differentiated C2C12 myotubes were measured. The results showed that the ROS levels were extremely low in the NC group and the D‐gal + NMN group. In contrast, D‐gal–treated myotubes showed obvious ROS‐positive fluorescence with substantially higher mean fluorescence intensity (Figure 3B,D).

Collectively, these findings demonstrate that NMN can scavenge the excessive accumulation of ROS induced by D‐gal and mitigate oxidative damage in senescent C2C12 cells.

### 3.4. NMN Inhibits D‐Gal–Induced Senescence of C2C12 Myotubes

**FIGURE 4:**
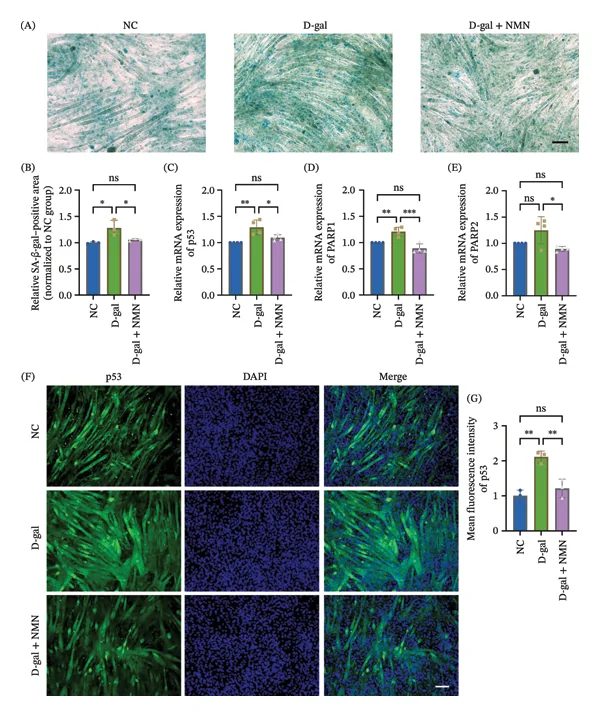
Effects of NMN and D‐gal treatments on senescence‐related phenotypes in C2C12 myotubes. (A) SA‐β‐gal staining of myotube cells after NMN and D‐gal treatment, *n* = 3. (B) Semiquantitative analysis of relative SA‐β‐gal–positive staining area. (C–E) Relative mRNA expression of senescence‐associated target genes, *n* = 4. (F) P53 immunofluorescence staining of myotube cells after D‐gal and NMN treatment, *n* = 3. (G) Quantification of mean fluorescence intensity of p53 in myotubes. Scale bars = 100 μm. All values were normalized to the NC group. All data are presented as mean ± SD; ns, *p* ≥ 0.05; ^∗^ *p* < 0.05; ^∗∗^ *p* < 0.01; ^∗∗∗^ *p* < 0.001.

Myotube formation is a necessary process for muscle function. To further verify the impact of NMN on D‐gal–induced senescence in myotube cells, the number of senescent cells was detected through SA‐β‐gal staining. The results revealed that D‐gal exposure significantly induced senescence in myotubes and elevated the proportion of senescent cells, whereas NMN intervention effectively reduced the number of senescent myotubes (Figure 4A,B).

NMN can also suppress the expression levels of senescence‐related target genes in C2C12 myotubes. After inducing senescence by adding D‐gal to the culture medium of C2C12 myotubes, the expression levels of *p53* and *PARP1* genes were significantly increased. Nevertheless, the expression level of *PARP2* gene showed no significant difference from that of the control group (Figure 4C–E). However, when NMN protection was added before inducing senescence, the expression levels of *p53*, *PARP1*, and *PARP2* genes were significantly reduced, showing no significant difference from the control group (Figure 4C–E).

To investigate alterations in the expression levels of senescence‐associated proteins, immunofluorescence assay results revealed that the mean fluorescence intensity of p53 protein significantly increased following D‐gal treatment (Figure 4F,G). However, the addition of NMN effectively suppressed the upregulation of p53 protein expression induced by D‐gal (Figure 4F,G). The aforementioned findings suggest that NMN can ameliorate the senescent state of myotube cells by modulating the expression of these senescence marker proteins.

### 3.5. NMN Inhibits the Expression of Senescence‐Associated Inflammatory Factors

**FIGURE 5:**
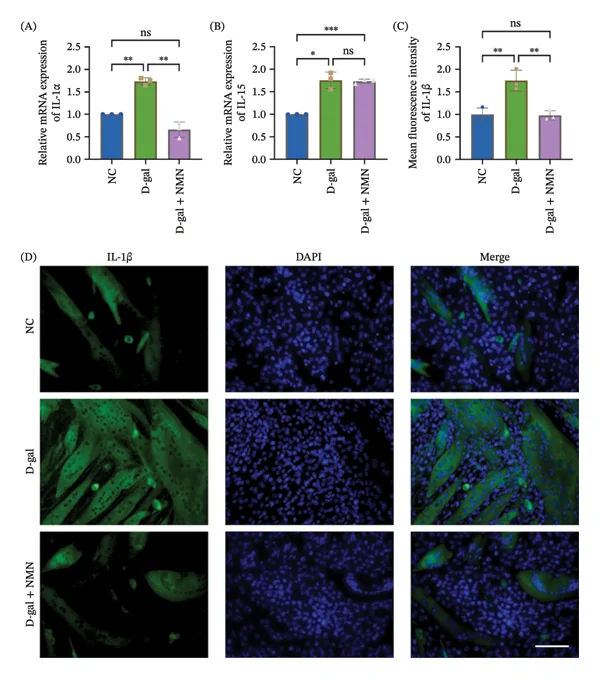
Changes in the expression levels of SASP‐associated markers in myotube cells after treatment with NMN and D‐gal. (A) Relative mRNA expression of IL‐1α, *n* = 3. (B) Relative mRNA expression of IL‐15, *n* = 3. (C) Quantification of mean fluorescence intensity of IL‐1β in myotubes. (D) IL‐1β immunofluorescence staining in treated C2C12 myotubes, *n* = 3. Scale bars = 100 μm. All experiments were performed in triplicate, with 3 random fields captured per sample. All data are presented as mean ± SD; ns, *p* ≥ 0.05; ^∗^ *p* < 0.05; ^∗∗^ *p* < 0.01; ^∗∗∗^ *p* < 0.001.

The accumulation of senescent cells and the increase in senescence‐associated secretory phenotype (SASP) factors can both promote the occurrence of senescence‐related diseases. In this study, the expression levels of SASP‐related genes were detected. The results showed that compared with the control group, D‐gal treatment significantly upregulated the expression of interleukin‐1*α* (*IL-1α*) and *IL-15* in myotubes. Pretreatment with NMN markedly suppressed the expression level of *IL-1α*, but exerted no significant inhibitory effect on the expression of *IL-15* (Figure 5A,B).

In addition, the expression level of SASP‐related proinflammatory cytokine interleukin‐1β (IL‐1β) protein was detected using immunofluorescence staining. The mean fluorescence intensity of IL‐1β was markedly elevated in the D‐gal group, and NMN pretreatment effectively suppressed this upregulation (Figure 5C,D).

## 4. Discussion

Aging can result in a decline in muscle tissue mass, strength, tone, and endurance, and D‐gal can induce an aged phenotype characterized by muscle atrophy in vitro [19]. In the present study, we established a cellular senescence model using skeletal muscle cells by treating mouse C2C12 myoblasts and myotubes with D‐gal. We found that NMN supplementation significantly alleviated D‐gal–induced cellular senescence in C2C12 cells.

Given that NAD^+^ concentration declines with age in human tissues, accumulating evidence suggests that NMN supplementation can restore blood NAD^+^ levels and may ameliorate age‐related pathological conditions [22, 23]. Research indicates that the age‐related decline in NAD^+^ levels is a key molecular characteristic of muscle aging and drives mitochondrial dysfunction [24]. Supplementation with NMN aims to reverse this trend, as NAD^+^ is an essential cofactor for longevity proteins such as sirtuins. Specifically, the activation of SIRT3 has been shown to attenuate high phosphate‐induced muscle cell aging [25], while SIRT5 protects primate skeletal muscle from age‐related deterioration by inhibiting inflammatory pathways through desuccinylation [26]. Our data demonstrate that NMN effectively restored MHC expression levels and promoted the recovery of myogenic differentiation in senescent C2C12 cells, suggesting that NMN ameliorates impaired myogenic differentiation in this cellular model. Integrating previous research with the results of the current study, our findings suggest that NMN may be a promising candidate for delaying muscle aging.

Muscle tissue is composed of muscle fibers, which originate from satellite cells and develop into multinucleated myotubes before maturing into functional muscle fibers in vivo [14]. In vitro, C2C12 myoblasts undergo myogenic differentiation and fuse to form multinucleated myotubes when cultured in differentiation medium. Aging induces impairment in myogenic differentiation in C2C12 myoblasts. Wang et al. reported that nobiletin prevents aging in D‐gal–treated C2C12 myoblasts by improving mitochondrial function [19]. We found that D‐gal–treated C2C12 myotubes exhibited atrophy, fragmentation, and sparse distribution of myofibrils. However, treatment with NMN at concentrations of 300 μM or higher effectively restored the normal morphology of the myotubes. Consistent with our findings, clinical trials have demonstrated that to achieve optimal therapeutic efficacy, NMN must attain a threshold concentration [23].

Aging skeletal muscle often manifests as arrested cell cycle progression and increased levels of ROS and SASP [27]. Under normal conditions, ROS is maintained at relatively low levels in the body [28]. However, upon physiological stimulation, ROS levels can increase sharply, exceeding the body’s ability to scavenge them, thereby causing oxidative stress damage [29]. Therefore, quantifying intracellular ROS levels is also an important indicator for assessing cellular senescence. By measuring intracellular ROS levels in myoblasts and myotubes, this study demonstrated that NMN inhibits the D‐gal–induced elevation of ROS, suggesting that NMN exerts a protective effect against oxidative stress–related cellular injury.

The p53 is activated in the early stage of aging and serves as a classic marker of cellular senescence [30]. Our previous research found that aging induces p53 expression in the dilator muscle tissue of the upper airway in mice [26]. P53 is involved in various forms of cellular senescence, including replicative senescence, DNA damage–induced senescence, and ROS‐induced senescence [31–33]. Increased p53 expression can induce skeletal muscle atrophy and accelerate muscle aging; conversely, the aging process can also upregulate p53 expression in skeletal muscle [33, 34]. During the aging process, DNA damage accumulates in the cell nucleus, leading to the activation of the poly (ADP‐ribose) polymerase (*PARP*) gene and the reduction of NAD^+^ levels [35]. In this process, the *PARP* gene participates in DNA damage repair in the body using NAD^+^ as a substrate [36]. D‐gal treatment significantly upregulated the mRNA and protein expression levels of p53 in myotubes, confirming that it successfully induced cellular senescence. Although D‐gal treatment did not uniformly upregulate all *PARP* family members, NMN pretreatment markedly inhibited the expression of *p53*, *PARP1*, and *PARP2.* This suppression reversed the cellular senescent phenotype, further substantiating the potential of NMN to delay muscle aging.

SASP is a major driver of tissue dysfunction and disrupted homeostasis in senescent cells. Its main components include inflammatory cytokines, chemokines, and other senescence‐associated secretory factors [37–39]. D‐gal induction activated the expression of IL‐1*α*, IL‐1β, and IL‐15, and NMN inhibited SASP expression by suppressing inflammatory cytokines IL‐1*α* and IL‐1β, thereby attenuating D‐gal–induced senescence in C2C12 myotubes.

## 5. Conclusion

The results of the present study demonstrate that D‐gal induces oxidative stress in C2C12 cells, accompanied by upregulation of senescence markers such as p53, DNA repair factors such as PARP1, and proinflammatory mediators such as IL‐1α and IL‐1β. In contrast, NMN significantly attenuated the senescent phenotype induced by D‐gal, highlighting its potential as a therapeutic strategy for combating muscle aging. Nevertheless, our current analysis is limited to proinflammatory components of the SASP; the effects of NMN on chemokines and matrix metalloproteinases warrant further investigation.

## Funding

This study was supported by Shanghai Science and Technology Commission, 22ZR1454300 and 19ZR1445500; Shanghai Stomatological Hospital, SSH‐2022‐KJCX‐B03; and Fudan University, FDUROP 254451.

## Conflicts of Interest

The authors declare no conflicts of interest.

## Data Availability

The data presented in this study are available upon request from the corresponding authors.
